# Attitudes and Perceptions of Health Leaders for the Quality Enhancement of Workforce in Saudi Arabia

**DOI:** 10.3390/healthcare10050891

**Published:** 2022-05-12

**Authors:** Majid M. Hejazi, Shayma S. Al-Rubaki, Othman M. Bawajeeh, Ziad Nakshabandi, Basim Alsaywid, Eman M. Almutairi, Miltiadis D. Lytras, Manal H. Almehdar, Maha Abuzenada, Halla Badawood

**Affiliations:** 1 Saudi Commission for Health Specialties, Riyadh 11614, Saudi Arabia; majedmhejezi@gmail.com (M.M.H.); shaymasaleh1@gmail.com (S.S.A.-R.); othmanmbawajeeh@gmail.com (O.M.B.); z.nakshabandi@scfhs.org (Z.N.); b.alsaywid@scfhs.org.sa (B.A.); e.amlutairi@scfhs.orga.sa (E.M.A.); m.almehdar@scfhs.org.sa (M.H.A.); m.abuzenada@scfhs.org.sa (M.A.); halla.badawood@gmail.com (H.B.); 2 College of Medicine, King Saud bin Abdulaziz University for Health Sciences, Jeddah 14611, Saudi Arabia; 3 Medicine Program, Batterjee Medical College, Jeddah 21442, Saudi Arabia; 4 Faculty of Dentistry, King Abdulaziz University, Jeddah 80209, Saudi Arabia; 5 National Center for Health Workforce Planning, Riyadh 11614, Saudi Arabia; 6 Saudi National Institute of Health Education and Research Skills, Riyadh 12382, Saudi Arabia; 7 Health Academy, Saudi Commission for Health Specialties, Riyadh 11614, Saudi Arabia; 8 Effat College of Engineering, Effat University, Jeddah 21551, Saudi Arabia; 9 Research and Development Center, Saudi Commission for Health Specialties, Jeddah 23343, Saudi Arabia; 10 Occupational Therapy Department, College of Applied Medical Sciences, King Saud bin Abdulaziz University for Health Sciences, Jeddah 14611, Saudi Arabia

**Keywords:** quality enhancement, health leaders, workforce competency, healthcare, perception, attitude, workforce planning, health transformation

## Abstract

Background and Aim: Besides the unique exposure and experience of health leaders in facing challenges and overcoming them, and the relatively fewer articles relating to the perception of health leaders in workforce quality enhancement, health leadership plays a crucial role in redirecting the workforce, increasing job satisfaction, professional development, and burnout prevention. Thus, this study aimed to understand the current healthcare workforce quality and future expectations from the attitudes and perceptions of health leaders. Methods: A qualitative research was carried out using semi-structured interviews consisting of 24 different questions. Participants of the study were healthcare leaders from different backgrounds and governmental institutions. All interviews were recorded, transcribed, and then analyzed using thematic analysis via the N-Vivo program. Results: Eleven participants were involved in the study, with one female and ten males. A thematic analysis and N-Vivo program yielded 5 main themes: (1) workforce competency, (2) health transformation, (3) leadership, (4) workforce planning, and (5) healthcare quality, with 22 emerging sub-themes. Moreover, participants responded with different attitudes and perceptions. Conclusion: Health leaders are satisfied with the current direction of workforce competency and planning, yet fragmentation of the system and poor accessibility may need further enhancement. Furthermore, misutilization of services and the uncertainty of the future and talent pool are potential barriers for capability building. Moreover, with the existing gap in the workforce, health leaders believe that privatization and corporatization may have a positive effect. Aside from that, Saudization with the current plan of having a minimum standard of accepting non-Saudis in certain areas might benefit in maintaining competition and enriching experience. However, catching up with further research in healthcare quality in Saudi Arabia is needed because of the ongoing health transformation.

## 1. Introduction

In Saudi Arabia, the healthcare sector has been growing rapidly over the past few years with 423,940 health employees as per the 2017 report by the Ministry of Health. With the adoption of the Kingdom’s Vision 2030, the healthcare sector is undergoing a powerful transformation approaching value-based healthcare [1]. With the change comes new challenges. An essential step to reaching long-term goals is effective workforce planning, a delicate combination of the science of analysis and the art of execution. Successful planning is initiated by laying down long-term strategic goals and correlating the current resources with the future workforce requirements, skill sets, staff levels, locations, and the costs of recruiting [2]. On the other hand, workforce planning alone is not enough. A competent workforce is crucial to maintaining the optimal quality of healthcare. Workforce competency describes the cycle of gaining knowledge and applying skills along with frequent reflection and feedback [3]. Identifying gaps in workforce competencies are beneficial for reconstructing educational goals and outlining desired outcomes. Ideally, healthcare education and practice must be interlaced smoothly where practice-based experiences should be included in academic programs while health practice should involve a more academic approach [4].

In addition, as the workload increases, it can become a detrimental issue for the workforce, affecting the quality of healthcare provided and practitioner well-being. According to Pastores et al., the increased workload is related to the severity of burnout among intensivists which can eventually result in employees leaving their jobs [5]. Furthermore, some studies predict shortages in healthcare staff in the coming years, the United States is expected to face a scarcity of one million nurses as well as a talent gap of up to 104,906 physicians by 2030 [5,6]. Moreover, the elderly population in Saudi Arabia is anticipated to rise from 3% in 2010 to 18.4% by 2035 [1]. With the increased demand for labor, it becomes easier for employees to look for new jobs, prompting employers to apply retention strategies with a special focus on the needs of the future employee [2]. For better health workforce planning, comparison with other countries with a similar health structure and employment was a sound method [7].

With the ongoing advancements in the healthcare sector, effective health leadership and management play a pivotal role in redirecting the workforce to efficiently respond to emerging issues. Leaders are constantly challenged to meet shifting expectations and set new priorities. Figueroa et. al. describes health leadership as the ability to recognize priorities, offer strategic guidance, and create commitments to achieve those priorities [8]. Thereby, successful leadership encompasses knowledge, skills, and the ability to engage with a different workforce [2]. Yet, a study done in 2019 in the United States mentioned that there has been little participation of healthcare leaders in sharing their perceptions of barriers associated with career optimization [9]. As mentioned by Martin et al., clinical supervision of health practitioners has been one of the well-founded ways to reach beneficial reflection from leaders, leading to increased job satisfaction, professional support, and prevention of burnout [8]. Considering burnout, it has been shown that about one-third of nurses and more than half of doctors complain of burnout symptoms [10]. Despite the profound knowledge of its impacts on the healthcare system, some leaders have little awareness about the ways to prevent it, ultimately bearing its consequences on the quality of healthcare. Therefore, leaders influence in order to improve outcomes on a wide range of aspects, especially when organizations focus on sustaining their patients’ compassion and well-being [11].

### Aim of the Study

In line with the aforementioned, healthcare workforce planning plays an integral role in fulfilling the Kingdom’s Vision 2030. On the other hand, exploring the health leaders’ attitude and perception towards the quality enhancement of the workforce is indispensable due to their unique exposure and experience in facing challenges and overcoming them. While there are numerous emerging studies with regards to workforce planning, relatively fewer articles are relating it with the perception of health leaders. The present study aims to understand the current healthcare workforce quality and future expectations from the attitudes and perceptions of health leaders.

## 2. Methods

### 2.1. Study Design

This study employed a qualitative, grounded theory design where we utilized predetermined semi-structured interviews to enable valid and reliable answers with in-depth responses. The semi-structured interviews were conducted in person and via an online platform depending on the ability of the participants and authors. 

### 2.2. Study Participants

Participants of the study are leaders of the healthcare sector in Saudi Arabia. A list was chosen purposively, and the participants were chosen based on availability. To ensure variability, leaders were chosen from different backgrounds and governmental institutions. Once contacted, each leader was introduced to the background and aim of the study, and consent was taken.

### 2.3. Study Setting and Data Collection

The semi-structured interviews were developed by the authors and reviewed by the principle investigator, and a pilot within authors was performed. The interviews consisted of 24 semi-structured questions, enabling openly valid and reliable answers. The semi-structured interviews were done between December of 2021, and January of 2022. 

The interviews were initiated with an introduction to the study and proceeded with consent for participation of the interviewees and recording the interview, after ensuring the anonymity of their identifiable information. Then, participants were asked different questions with regard to the workforce, health transformation strategy, leadership, workforce planning, and the quality of healthcare. 

### 2.4. Data Analysis

Interviews were recorded and then transcribed according to each question. The study conducted a thematic analysis using the N-Vivo program. 

## 3. Results

During the initial phase of our research that investigates the attitudes and perceptions of healthcare leaders for the quality enhancement of the workforce, we wrote 24 semi-structured questions, and we came out with 5 themes, and a total of 22 emerging sub-themes. Each theme has a focus on the issues of each area in the workforce enhancement. The first theme discusses topics related to workforce competency; the second talks about the health transformation and its relation to the workforce; the third focuses on leadership, from qualities to proposed enhancements; the fourth theme discusses workforce planning, regarding its importance, areas of exploration, change management strategy, and workforce upskilling; and, lastly, the fifth theme examines the healthcare quality area. Overall, 11 healthcare leaders participated in the study. One participant is a female, and the remaining ten are males. Each participant was assigned with a hypothetical name, which are Dr. Ahmed, Dr. Abdulaziz, Interviewee #11, Dr. Ali, Dr. Hamed, Dr. Salem, Dr. Bandar, Dr. Yazan, Dr. Adnan, Dr. Fatima, and Dr. Saleh (see Table 1 below).

### 3.1. Workforce Competency Theme

This theme expresses the attitudes and perceptions of the research’s participants over the presenting status of workforce competency, barriers that were faced or could be faced with regard to workforce capability building, and different ideas and suggestions for the enhancement of workforce competency. Thus, three emerging sub-themes were identified. 

#### 3.1.1. Current Status of the Workforce

##### Competency Sub-Theme

When the interviewees were asked about their perceptions of the current workforce competency, we noticed several positive feedbacks. Starting with Interviewee #1 when he said, “All our education is competency-based”. He further explained his point of view with “We see the quality indicators in hospitals and our Saudization percentage is very high”. Moreover, Interviewee #2 said, “I think we have made great progress when it comes to the workforce competency”, and then he discussed his claim further, and said, “When you look 50 years back, we barely had a Saudi physician who is qualified to deliver any healthcare service at all. However, currently, we have almost all the specialties that you can think about here in Saudi Arabia”. Additionally, he stated that “Qualifications and competencies are measured and monitored with the best standards. Saudi Arabia now is one of the best five”. Interviewee #3 stated positively about the workforce, and said, “I think the Kingdom has one of the best workforces in the world from the perspective of quality and quantity”. Moreover, Interviewee #4 talked generally on how there is always room for improvement, but discussed a positive concept, and mentioned, “Workforce competency as you know is always something where there is scope for improvement. I would say that in terms of competency we could compare ourselves with other countries in the world and I would say as a G20 nation we have much better competency compared to other nations”. Similarly, Interviewee #5 said, “In terms of competency, I think there are areas of gaps. However, I am very pleased with the level of competency compared with 5–10 years ago”.

Some of the interviewees mentioned gaps that need to be addressed. First is with Interviewee #6 when he said, “I think workforce competency needs to be improved always, especially when we shift our paradigm from income to outcome. When we do this shift, we will have lenses that can show workforce competency”. Moreover, he stated that “What we are doing now in our transformation is focusing on the outcome. I think that our situation now is not elevated to our passion, our passion is to have a workforce with high quality”. Meanwhile, Interviewee #7 mentioned the progress and gaps and said, “I think we have passed through a good range in building up the capacity of the workforce in the healthcare sector, and there are a lot of gaps”. He also mentioned that ”I think there is a gap even in the medical and surgical staff, but I think the gap is much higher in allied health and nursing with and an existing gap in the medical”. However, Interviewee #8 talked about this from a broad perspective, and said, “The workforce should be viewed through the different clinical specialties”. Moreover, she stated that “When we are talking about the workforce we are talking about knowledge, skills, and attitude. It’s variable from one specialty to another and varies from hospitals and sectors. I would say there is a big room for improvement”. Lastly, Interviewee #9 mentioned several challenging aspects and said, “Attracting the right people, bright talent is one issue or challenge, the other thing is providing a template for training, upgrading, and upskilling of the workforce to meet the required competency is another challenge. As well, given the transformation, there are various work roles that the market does not have enough workforce to meet these competencies and these needs”.

#### 3.1.2. Barriers of Capability Building of the Workforce Sub-Theme

There are many barriers mentioned by the interviewees, wherein three participants mentioned the system as one of the barriers. Interviewee #6 said, “I think yes, we have fragmentation of the system regarding workforce planning. The fragmentation of the system is the main barrier because we have 8 healthcare providers and the private sector, they are independent systems”. Additionally, Interviewee #10 said that the system is complex due to the workforce coming from different backgrounds and working in different places, wherein he mentioned, “The complexity of the healthcare system, in general, makes it quite challenging to work on the different levels required and different skills. So, I think there are challenges for sure”. On the other hand, Interviewee #7 said, “I think there are a lot of potential opportunities that are not being utilized properly. We have a unified system for education and postgraduate training, so that is an opportunity like we are not a fragmented system as you will think in other countries and other opportunities that are not utilized properly. Once you have a unified system, make the proper analysis and diagnostics, then you will be working on a solution to treat these issues in the private sector, public sector, charities, and all that. There will be some variations. I don’t see them as a challenge. I see the challenge is not doing what is supposed to be done”.

Regarding the services, Interviewee #8 mentioned the effect of unutilized services on training, and responded, saying, “The most important part is to have fully utilized services, but if they do not have the utilized services, they will not have enough cases and that would affect the training. So, this is the biggest barrier”. Furthermore, Interviewee #4 spoke about the uncertainty of the future and said, “You can build capacity for what is an expected path of growth, you cannot build capacity for emergencies. What we can do is we can bring agility for emergencies, or resilience”. He also added, “The barriers as I see them, we have a workforce barrier in terms of the talent pool that we have to use in the workforce, and that fits into almost every other aspect of workforce competency”. Lastly, Interviewee #5 pointed out the fact that we have limitations in job opportunities and the incoming pool, offered a way out, and said, “We need to have employers that are willing to accept entry-level expertise”.

#### 3.1.3. Enhancement of the Workforce Competency Sub-Theme

Two of the research interviewees mentioned the current efforts that are taking place. Interviewee #5 stated that there is a current task that is being carried out, and, afterward, he pointed out an idea for the continuous improvement of the task, so he said, “The Saudi Commission has a close relationship with this task through the expansion in between the programs that it imparts on in the past few years as a part of its strategic plan. Also, through the establishment of the health academy that is directed to address this skill gap in the workforce”. He further mentioned that “We have to have numbers to measure whether these are adequate initiatives or we need to do more”. Meanwhile, Interviewee #2 mentioned that enhancement cannot be made, and he pointed out certain areas are being utilized and said, “Enhancement is always going to be impossible because no matter what you do, there is always going to be room for an enhancement. Now enhancement is on two levels, which are clinical and technical skills, and the efforts for these are minimal to moderate. However, admin skills are completely absent, so we are trying here in the leadership academy to create opportunities for this kind of training; creating frameworks; admin programs; leadership journeys; and digital platforms so people can train themselves. There is a lack of competency in the organizational structure which includes HR functions, talent management, succession planning, strategy implementation, and other things that should be in the healthcare system, and that is the reason for building workforce planning because it is needed to fill that gap”. Interviewee #8 talked about the importance of training and what the Saudi Commission is doing regarding it, and said, “We need to have very good training. So, we need to improve it in quantity and include more hospitals to be part of the postgraduate training, and we need to qualify more trainers within those hospitals, which is what the Saudi Commission of Healthcare Specialties is doing”.

The other participants raised several points for the enhancement of workforce competency. For example, Interviewee #7 raised the idea of doing comprehensive data analysis and said, “I think we should run a comprehensive data analysis when it comes to competency and run this gap with all vast healthcare and education from universities, colleges, Saudi commission, and hospital training. Once we do that gap analysis, from my perspective, I am sure that others will share their concerns and try to have a unified comprehensive solution that engages undergraduate and postgraduate education and training”. Next, Interviewee #1 raised an opinion about the consistency of the competency and said, “Recertification is one of the things that would guarantee, after a longer period, that we still have the competency to practice”. Moreover, Interviewee #4 mentioned three points of intervention for enhancement: the first one is checking the workforce at the point of entry, the second one is after graduation, and the third is constant learning. Interviewee #9 stated that “If you have well-known standardized competency, you can develop a road map towards closing these gaps that will influence your training programs, the resources that you need to close these gaps in terms of training requirements and others”. On the other hand, Interviewee #10 explained a unique idea, and said, “I think we have to involve really good standards in place where we are testing our students even before they graduate from internships and not leave it to the hospitals where they train”. He also added, “Anyone who graduates should have a competency assessment by the Saudi Health Commission rather than relying on the fact that they just graduated from a Saudi university because the outputs of these universities are very vast and different and I think that we could always tap into scientific committees that exist and as well as scientific bodies that are independent that can help develop these competency assessments for the students or the applicants coming into the workforce and also ongoing assessment of competency”.

#### 3.1.4. Key Findings and Interpretations Related to the Workforce Competency Sub-Theme

The well-organized overview of key opinions and attitudes of the Leaders related to the workforce competency sub-theme provides fertile ground for key interpretations. In Table 2, we provide an initial summary and interpretation of the key findings. 

According to the key facts related to the opinions and the attitudes of Leaders, these are some key statements.

**Strengths/Facts:**

There is a common consensus of Leaders that the current assessment of the workforce competency in Saudi Arabia is positive. According to our interviewees, the most significant aspects of this include:

- The Competency-based Medical Education system in KSA supported by various activities also of the SCFHS.
- The continuous measurement of Quality in Health Education and Training and the adoption of top international standards.
- The objective assessment of the Saudi Workforce as one of the best in the world.
- The excellent perception of the current status of the health workforce in terms of competency.

**Areas for Improvement/Gaps:**

The interviewees in our research overall have a very good perception of the current competency and efficiency of the health workforce. They emphasized, though, that focused initiatives for improvements are required, most of them associated with well-identified gaps that exist is medical and surgical staff and also in allied health and nursing. The most important areas for improvement according to their statements are as follows:

- The need to continuously update the knowledge, skills, and attitude that varies from one specialty to the other and from one hospital to the other.
- The great challenge of attracting the right and bright people in terms of talent and attitude.
- The importance to provide a standardized and customized and template for medical education and training.
- A systematic, inspired effort for upgrading and upskilling the workforce.
- The challenge to meet the impact of transformation that generates new workforce specialties and opportunities given the lack of workforce in these areas.

**Barriers:**

The current assessment of the Health Workforce by our interviewees also highlighted significant barriers that need to be understood further to provide revisions in our current approach. The most significant barriers are summarized as follows:

- The fragmentation and the complexity of the system.
- The unutilized services.
- The uncertainty of the future and the need to bring agility for emergencies or resilience.
- The current existing limitations in job opportunities and the incoming pool of jobs and competencies.

**Challenges/Opportunities:**

According to our interviewees the challenging unified system for education and postgraduate training in Saudi Arabia is a great asset and one of the greatest opportunities in a novel, integrative approach for solutions that will involve the private sector, public sector, and charities.

**Enhancement:**

Our participants also commented on the key areas for possible enhancement in the current workforce competency. They emphasized on the positive impact of current, undergoing initiatives for enhancement and of the implemented strategy in the recent years in the SCFHS. Additional facts include:

- The Health Academy is a key enabler of value delivery.
- Enhancement related to clinical and technical skills is required and must be a priority area.
- Administrative skills are absent and need enhancement. Within this context, special focus must be paid on creating frameworks, admin programs, leadership journeys, and digital platforms.
- There is a lack of competency in Organizational Structure including (HR functions, talent management, succession planning, strategy implementation).
- There must be a continuous effort and focus on the enhancement of the Quality of Training and the availability of top-quality trainers.
- A comprehensive data analysis related to competency gap with all vast healthcare and education from universities, colleges, Saudi commission, and hospital training must be undertaken. This will lead to a comprehensive solution that engages undergraduate and postgraduate education and training.
- Recertification is a bold initiative to secure competency.
- Continuous assessment of workforce competency and need for a standardized competency framework that will inform a roadmap for development.

### 3.2. Health Transformation Theme

Health Transformation is a theme that discusses the perceived components of health transformation, the understanding of each participant leader in the concept of value-based healthcare, the possibility of new emerging roles that could be of aid to the healthcare transformation strategy, the incorporation of non-clinicians as part of the healthcare workforce, and privatization and Saudization in healthcare transformation strategy.

#### 3.2.1. Components of Healthcare Transformation Sub-Theme

Starting with the definition of healthcare transformation and the way it started, Interviewee #2 emphasized it as a major component in the vision for 2030. Then, he explained how it started and said, “It was a small part under the life quality stream, but now it has been upgraded and became its program, a dedicated one for the health transformation”. He then added three essential points of health transformation and stated, “First, economically, the outcome is not equal to the governmental spending, so we need to adjust this cost-effectiveness issue. Second, if we want a very healthy community in 10 years, everyone should have access to healthcare regularly. And, third, healthcare services should be improved and upgraded in all regions, and without this, it is going to be very difficult to have health transformation in the entire kingdom”. Similarly, Interviewee #5 stated that “Right from the beginning of the vision realization plans that started with the vision 2030 back in 2016, there were five main directions where transformation was needed. It consists of access to service regardless of where they live, who their employer is, and their age and social status; the quality of service; quality of care delivery; improving financial efficiency; reducing the burden of chronic diseases; reducing the impact of accidental injuries. If all of that is employed properly, they will fulfill the national ambition of a prosperous society with higher life expectancy and healthy life”.

The other participants discussed the components of the healthcare transformation. Interviewee #9 mentioned the core of the transformation, saying, “The core of transformation is to move from the model that is based on the hospital and institution to more around the center, around the beneficial. The second thing is to shift our attention from healthcare to prevent illness so work upstream on social determinants of the north to create sustainability which is one of the objectives of the transformation”. Interviewee #4 stated the requirements of achieving the transformation and said, “We need to have better health, better care, better value, and reduce accidents… These are some of the objectives that the healthcare sector has. Value-based healthcare is another thing that benefits everyone and improves systemic efficiencies”.

Regarding the challenges, Interviewee #1 mentioned several ones and said that “That is why the whole transformation takes from 10 to 30 years, it is a healthcare system, it is not easy, especially that transformation is happening while the system is running. You cannot stop the healthcare system to apply the transformation strategies and that is why it is very complex and not easy. And we are being challenged to provide the best service with the highest value in the least possible time and this is what we aspire to. Because our issues are unique, we can learn from other people, and we can provide amazing solutions that no other country has approached previously. Our leaders tell us that nothing is off the table, just make your case and go ahead”. Meanwhile, Interviewee #7 brought up the progression of this transformation, saying, “I think when it comes to the vision 2030, it has been unfolded with a model of care. The new system with Public Health Program (PHP) to purchase and fund healthcare services. For privatization, there are a lot of things that are happening and have unfolded before us. We are part of this transformation, we are the enablers to this transformation, we have the national program that was started in 2018–2019 and we are moving to phase 2 right now”.

#### 3.2.2. Concept of Value-Based Healthcare Sub-Theme

The interviewees were asked about their concept of value-based healthcare, and all of the interviewees explained the concept in a unified way, except one participant. To illustrate what the participants said regarding their unified perception in value-based healthcare, Interviewee #5 said that “Minimizing cost, and maximizing value are the main drivers of value-based healthcare”. Likewise, Interviewee #6 said, “It’s a concept to build our payment to the outcome. This is the main idea by focusing on the outcome instead of focusing on input or tools which is the main goal of going with that paradigm. Value-based healthcare is the future of healthcare quality and services”. Moreover, Interviewee #4 explained it as ”Value is defined as outcomes by cost. The outcomes that you desire through delivering healthcare as a service. The outcome when it is divided by the cost. One way to understand value-based healthcare is to compare it with what exists today. In many places we don’t have value-based healthcare, we have a fee for service, where you look at a transaction as an episode. A person with a headache goes to the hospital, the doctor checks them, orders tests & scans, and the headache is treated on an episode basis in the sense that the patient will go back to the doctor when they have another episode of headache. So this is episodic, where the doctors or healthcare providers are reimbursed based on the fee for service they get. When you look at the value-based concept, what they measure is the outcome, there is a big difference there”.

On the other hand, Interviewee #3 talked about it differently, saying, “This is interesting because value-based healthcare depends on who is assessing the value, as the English example says, beauty is in the eye of the beholder. So the value to who, value to the government, to the nation, to the patient, or the physician or practitioner. This is very important but let me prioritize. I think that value to the patient and value to the nation are the two most important that we need to look at. For sure, the value to the practitioner is still very important, just to get it out of the way. Value to the practitioner means that is it better for me to spend my time trading stocks or practicing the medicine of any kind, is it better for me to be an engineer or pilot or a healthcare practitioner”. Moreover, he explained from the patient’s view and said, “If you come from the patient’s perspective regarding the value, they would like all the beds and all the services to be like King Faisal’s Hospital or America and the reason why they would want this is so simple because they do not look at the cost, so the equation of value is disturbed. The reason why they picked King Faisal or America is that they are looking at the outcome and not the full process, where how much you put into the system and what outcome you get out of it”. In the end, he mentioned his concern, saying, “I’m worried that there might be confusion in these subjects among leaders and that’s my concern”.

#### 3.2.3. New Roles in Healthcare Transformation Strategy Sub-Theme

The interviewees mentioned how important it is to have new roles and how they are going to aid the achievement of this strategy. Interviewee #2 mentioned what we need and the current activity of the health academy regarding this point, saying, “It depends on the strategy that is built for the transformation. So, for the regulators, we will need policymakers. For corporatizing, we will need strategists, audit, and risk managers. For the pair system, we will need health economists. Therefore, each strategy is going to assign new roles that we might need. Now the health academy is doing a program for 29 new roles for this transformation”. Meanwhile, Interviewee #8 emphasized the importance of some new roles supporting the vision for 2030, responding with, “If you look at the vision of 2030 goal, it states that we need to increase the average life expectancy from 74 to 80 by the year 2030. Now this is a very ambitious goal and for that to happen, not only do we need to increase the existing specialties, we have to be thinking about new specialties like genomics, people that specialize in Artificial Intelligence (AI) in healthcare, and support management are important. Therefore, several areas need to be included and I think that is why having a national healthcare center of the workforce for planning is going to be a very important catalyst to help us to realize the cause of transformation”.

Interviewee #4 talked about artificial intelligence purely, and stated that “We have to understand that when we look at artificial intelligence, there is some amount of artificial intelligence at play today. There is what we call mission learning, the artificial intelligence that we are using, it is everywhere including in the healthcare sector. Robot technicians are there, but very sporadically, and some surgeries use them. If you look at laser surgeries, they are largely robotic. But in terms of replacing human labor, these technologies are slightly far off, and I say this because human beings are capable of general intelligence. The same human being can wash a car, cook food, walk a dog, and much more. Today what we have in artificial intelligence is what we call artificial narrow intelligence”. Moreover, he mentioned that “New roles can come up when the telemedicine network becomes bigger and as said, robots and artificial intelligence are going to play a role in that scenario”. Additionally, Interviewee #9 mentioned some support services and said, “Of course, there are gaps in nursing and that needs to be addressed, that goes without saying. However, we need a lot of support services, such as case managers, coordinators, health coaches, we have a dire need for coders which is one of the most painful areas right now in the system. The rate of training of coders and the number of trainees produced annually is very low and will not meet the need at all the number of primary healthcare or family physician trainees are coming more than coders”.

Interviewee #5 mentioned that we need new roles, but to determine them, there is a certain process that has to be followed, so he said, “there are many new jobs and new skills that will be needed. However, to say what exactly they require proper analysis and study. Whatever role that is going to be created has to come out of a study and assessment that has originated from our environment”. On the other hand, Interviewee #3 emphasized the importance of reconsideration of the current jobs, and he responded, saying, “Many new roles will be required, and some of them are directly related to innovation in medicine and some are related to the vision itself. We need good health economists, people who understand the different policies worldwide, people who speak different languages, and we need technology, data, and revolution. When we transform from treatment to prevention, we will need more people for prevention. We will have to rethink and reprioritize the jobs we have now” Conversely, Interviewee #7 mentioned that there is no need of creating new job titles from a specific point of view, saying, “I think that we have what is required without even creating new titles but to a certain degree having a new title would probably reinforce change because right now the jobs that we have right now are not doing the most properly”. To a degree of similarity, Interviewee #9 said, after emphasizing on the support services, that “I think we need training, not as much as roles, but we need to train leaders to be able to steer and manage this transformation”.

#### 3.2.4. Incorporation of Non-Clinicians as Part of the Healthcare Workforce Sub-Theme

Starting with the emphasis of the importance of incorporation of non-clinical encounters as part of the healthcare workforce and its current direction, Interviewee #3 said that we already have non-healthcare workers that are working in healthcare, then mentioned a concern of his, and said, “If these workers do not understand a thing in healthcare, it would be a problem. Therefore, we need to ensure that those people are well trained and ready to work in the healthcare sector”. However, Interviewee #7 spoke with regard to the aforementioned issue, and his response was that “There is good education and training that is required to bring them in the capacity to fill in an important role in a chain of care of the patients”. In addition, Interviewee #4 stressed the relationship of non-clinicians with preventative healthcare, stating, that “If you look at the emphasis we have put on preventative healthcare as a part of the transformation, they are largely non-clinical encounters. Everything that fits within the bowel of preventative healthcare could be non-clinical”.

Regarding the areas that are needed, Interviewee #9 mentioned several ones, and said, “We have a tremendous need for non-clinical services and experts, that will address the need that concern costing, coding, data, information, and there are a lot, but these are the primary non-clinical roles that will need a tremendous work on in terms of closing the gap”. In addition, Interviewee #6 recalled various examples, in which he stated, “Non-clinicians are important, they are enablers for the healthcare services, such as financial people, legal, human resources (HR), and administers. All these people are enablers for the healthcare services”. Then, he suggested a process to follow, “We have to expand our plans to know that there are some fields that we need to focus on that can help our healthcare build on; means we have to have a very wide plan for the workforce as an infrastructure workforce to build high-level services in healthcare”.

#### 3.2.5. Privatization and Saudization in Healthcare Transformation Strategy Sub-Theme

The study participants were asked about their own opinion with regard to privatization and Saudization. At first, Interviewee #2 argued about the interrelation of Saudization and privatization, saying, “Privatization and Saudization do not go hand in hand because private companies have their requirements and usually focus on low market price, and unless we have a great workforce plan, we might slip and get back to where we were and then Saudization drops”.

Then, speaking with regard to privatization, Interviewee #1 talked about the meaning and importance of privatization and said, “Privatization means that you corporately run things like other companies. It is a practical way of separating legislation from implementation. It helps to create value. And a system that is based on value and has specific clear goals helps to make things more efficient and productive”. Similarly, Interviewee #9 explained the objective and his correct meaning of the privatization that will be worked on in the health transformation by saying “Maybe there is a misunderstanding happening right now in the transformation. This is not privatization, this is corporatization. So, the clusters are not being privatized; they are not going to be owned by anyone other than the government. The government has full ownership of these clusters and their chief executive officers (CEOs), but they are going to be corporatized. That is what we call institutional transformation" Further, he explained what corporatization will lead to, and said, “Corporatization will create this level of transparency and accountability because, at the end of the year, you need to be accountable in front of regulatory bodies, including ourselves”. Additionally, Interviewee #7 spoke with regard to its meaning from the view of the participation of the private sectors and said, “Privatization is a privilege; when we are privatized, you get more money, and that will increase the margin of interest, which is not the purpose of privatization. Privatization is to forcefully engage the private sector, that is at least what I am seeing, engaging the private sector in increasing Gross Domestic Product (GDP) and in partnership with the government and, at the same time, you are looking at them to improve the quality”. Moreover, Interviewee #5 gave a simplified explanation of the importance of applying privatization in the right way, saying, “It could affect it positively and negatively. We have to realize that potential. We have to direct it to the benefit of closing the workforce gap in a proper setting”.

Regarding concerns, Interviewee #7 mentioned his concerns regarding privatization and what we might face in the participation of the private sector: “I think it depends on how it is done and we do not add a lot of knowledge of how it has been done, privatization wise. However, I think the worst thing and the most harmful thing is looking into privatization as something we must achieve, and I think it is an issue for the private sector to think that it is just something that we have to do to get more money. I think this is the worst thing that could happen because you know with privatization comes mandates, comes a serious responsibility, and accountability”. Meanwhile, Interviewee #6 explained the purpose of corporatization and a potential big obstacle that could be faced, stating that “The main purpose is to have a flexible system to change the traditional way of compensating healthcare professionals and focus more on outcome and performance, not only fixed salary, and creating a system for incentives for rewarding and promoting. With corporatization, you will have that flexibility more than traditional services. The civil service system is a big obstacle to improving our health workforce. Here in Saudi Arabia, we have a unique concept of privatization, including means in our context. Privatization includes corporatization”.

However, Interviewee #3 mentioned the goal of corporatization, and stated that “Privatization is not my ultimate goal, but corporatization is; which its goal is to know the value chain of the whole money flow from the first minute to the last. When we get to that, we will be more competent”. Lastly, for privatization, Interviewee #8 talked about the experience of other countries and mentioned the importance of the regulator in privatization: “If you look at what privatization did in other countries, there are mixed experiences and successes, and there are some challenges as you are doing privatization, which is very important in terms of healthcare provision and making sure that we have an efficient healthcare system. You want to make sure that the regulator part is strong. If I am getting a lot of services from the private sector, I have to make sure that the regulator within the minister of health is capable of oversight”.

Regarding Saudization, Interviewee #5 explained the importance of Saudization and its impact on society and the health transformation: “It is an ultimate goal, and it will affect the healthcare practice in the form of more stability in the workforce, in the form of satisfaction of the population that does not speak foreign languages. Therefore, it will for sure come with its positive impact, but it is not without a price; it has to be invested on, and thought about in a longer-term plan”. Interviewee #9 spoke with regard to the reasons behind this movement and said, “I think that, in the end, Saudization is a strategic objective for the country. So, we have no option but to go in that direction for various reasons. One of them is security, and another one has to do with using local talent and local content”. However, Interviewee #1 spoke about how challenging it is to be achieved and said, “Saudization is the holy grail. The challenge is that we are expanding in certain specialties, after a while we will need to close down certain specialties due to reaching saturation”.

Despite that, four of the participants raised a similar opinion to a certain degree, as Interviewee #2 mentioned, “I do not think we should go 100% Saudi, there has to be a certain percentage of international experts who are in the system so we can enrich the experience”. Interviewee #8 further elaborated, “For any country, the nationalization of the healthcare services is a nationalization for the workforce, in general, is important. 100% nationalization is something that cannot be achieved in any healthcare system. If you look at the advanced healthcare systems in North America and Europe, they always have a diverse workforce, so I think the goal is to add as many Saudis as possible to the system. However, at the same time, you want to make sure that you are going back to the value-based healthcare that you do not do at a pace or speed that would end up having people that still not be qualified, and that would have an impact on the quality and value-based healthcare”. Meanwhile, Interviewee #6 said, “We must create a safety plan and a secure plan for the country to have local people and there is a plan to have a minimum standard of accepting non-Saudis in some areas”. In addition, Interviewee #3 said, “If my goal from Saudization is something from nationalism, then it is something different, but what I want is stability in the work environment, and that is what is important. So this Saudization movement needs to achieve work stability and to maintain competition between Saudis and non-Saudis”.

### 3.3. Leadership Theme

In this theme, the participants of the study discussed two topics that emerged as sub-themes. The first topic that was opened is the current leadership qualities of the healthcare sector, while the next one was about their perceived suggestion of enhancement of certain criteria of the employment of healthcare leaders. 

#### 3.3.1. Current Healthcare Leadership Qualities Sub-Theme

Qualities of healthcare leaders have been described to be of great descent by four of the interviewees. Interviewee #1 stated that “No one takes an administrative position until and unless they are eligible or have the background to do it”. Similarly, Interviewee #2 said, “They are leading the transformation and they are holding huge responsibility and accountability. I believe they are the best”. Another comment to add is Interviewee #4’s emphasis on the importance of distributive leadership, and then his own opinion of its presence: “When you say leadership, one of the things that the healthcare sector needs are distributive leadership. In the sense that any person in the position of a decision-maker is a leader. So that person could be down the line, at the top or any place, and especially in complex situations like the pandemic, you need leaders and decision-makers at all levels. If you look at the pandemic which is the most recent situation we can relate to, I can say we are on the right track”.

On the other hand, six of the interviewees demonstrated a certain degree of concern, as Interviewee #10 mentioned, “Leadership happens because of seniority rather than skill,” and he also added that “Now most of the work being done is about system thinking, complex theory, empathy, emotional intelligence, building teams, and getting the best out of your teams, but I don’t see that a lot. It is rare to see these skills in the actual workplace. You hear people preaching about them, you hear people presenting about them, but when it comes to day-to-day work, it is not something that we see”. Moreover, Interviewee #3 emphasized three points, saying, “We need more skills for this rather than just scientific skills and knowledge. Also, there is a wrong culture that we have, which is that those who are older are better at leadership, and this needs to be changed. Healthcare leadership is sadly not present”. Furthermore, Interviewee #8 compared leadership with clinical programs and said that “We have fully developed clinical programs, but when it comes to leadership, we just assume that someone good clinically is also good as a leader. Therefore, when we want to put someone as a leader, they have to be fully developed and certified into leadership”.

Apart from positives and negatives, Interviewee #10 and Interviewee #6 suggested ideas. Interviewee #10, after mentioning the negative point above, said, “And also investing in the younger population might allow us to discover emerging leaders we had no idea could be invested in and be the future leaders of the organizations”. Interviewee #6 said that “We need to develop leadership into the new concept of a learning organization and adaptive leadership”. 

#### 3.3.2. Areas of Enhancement in the Criteria of Leader Employment Sub-Theme

The complexity of the approach of enhancement in the criteria of leader employment was demonstrated by three of the interviewees, as Interviewee #10 explained, “We might have the best program for a certain specialty, but the outcome might not be what we are looking for”. He also suggested that “We have to invest in leaders, leaders who work as teams, and who understand the ultimate purpose”. Meanwhile, Interviewee #7 said that “This is a very complicated question to answer,” with a general emphasis saying, “I think we are lacking proper planning and proper execution and I think we need more education and training toward these aspects”.

Talking about qualities, Interviewee #1 mentioned that “Main thing in leadership is that they have to be brave, to collaborate, and be open-minded because they will not succeed alone, and that is the key”. Additionally, Interviewee #3 discussed the qualities, in general, and then, specifically, in the medical field, saying, “Too many. What I expect from a leader is social skills, team building & enhancement, justice, fairness, and communication, nonetheless of what area this leader is going to lead. However, when it comes to a specific specialty, like medicine, they must have a medical background. In addition, leaders before they become leaders need to be trained and supervised for a certain time”.

On the other hand, Interviewee #5 mentioned a special way with regard to training instead of criteria enhancement, saying, “If they start right from the beginning where healthcare practitioners start in their graduate training, that point has a major role to implement leadership capabilities right in there because most healthcare practices require some degree of leadership skills, so that is where things should start. Then, providing opportunities for guided leadership in healthcare provision settings in a nonthreatening and safe environment. Also, to allow for the system to bring that culture where leadership is encouraged”. Interviewee #11 emphasized the current implementation of leader employment and said, “So far, we have done so many assessments within the healthcare system about the acquired skills. For example, those who are versed in finance, strategy building execution, good communication skills, and who puts the patient at the heart of the process. One of the first things that we have done here in the leadership academy is that we have built a model that has two core competencies or values which are accountability and care, five competencies and eighteen behaviors that we should develop in leaders and that was based on many workshops with healthcare leaders in the Saudi health sectors and it was reviewed by organizational psychologists. So, every program that we developed was based on this model”.

### 3.4. Workforce Planning Theme

Workforce planning was the focus of this theme and discussed several matters, including planning strategies, examples of adopted strategies, the required exploration of certain areas of planning, the golden rule of each participant of workforce planning, determining the needs to meet the vision for 2030, change management strategy, workforce upskilling, and the attractiveness of healthcare ecosystem to the workforce.

#### 3.4.1. Importance of Planning Strategies Sub-Theme

When participants of the study were asked about the importance of workforce planning, all of them agreed on its importance. Interviewee #6 emphasized its importance and said, “Workforce planning is the first function that the regulator must provide”. Furthermore, Interviewee #8 explained its importance on health transformation and said, “If we want to improve life expectancy from 74–80 by 2030, as one of the items of health transformation, we need to have a specific number of workforce categories and a specific number of individuals, we need to look at them through different regions to make sure we do not leave anyone behind, how many we need from the outside, and how we can leverage technology on our side. Then, you can make sure that people are receiving promotive, preventative, and curative healthcare services when they need it”.

Additionally, Interviewee #2 explained what would happen when it is not done properly, saying, “When a post-graduate gets into the market, many of them have difficulties getting accepted because of many specialties being very difficult to get into and that is because of faulty planning. You have under-graduate training and post-graduate training capacity, if this cycle is not in continuous coordination, you end up with people who are highly qualified and skilled without jobs. And also, you will find unmet needs and some regions that do not have the wanted specialties”. On the other hand, Interviewee #4 related the question to countries abroad and suggested a plan, saying, “Because there is a reliance on the workforce from outside the country, our strategy should extend beyond the country. Some investment has to be made by tying up with an international institute or college to say that we are happy with the graduates that come out of this college and we want to recruit them” Furthermore, Interviewee #3 explained the issue with workforce planning after emphasizing on its cruciality, and said, “The problem with workforce planning is that it depends on certain inputs or variables, like the socioeconomic status and diseases that are common in the community. Also, the tools we use are not fast ones”.

#### 3.4.2. Examples of Adopted Strategies Sub-Theme

After asking about an example of an adopted strategy, four of the interviewees gave negative feedback. One detailed comment is Interviewee’s #3 where he emphasized the fragmentation issue and said, “There are workforce planning strategies that are being deployed by different sectors within the health sector in the kingdom. We do not have only one sector, but we have 7, and each sector is planning alone and each one’s priorities are within their priorities and entity and not within national priorities. Therefore, yes we have plans, but we hope that there are no plans like these”. Likewise, Interviewee #4 mentioned an external example reflecting the power of unity, saying, “If you ask Uber how many cars are running in Riyadh, how many are moving, and how many are idle, they will be able to give me all the numbers because their system is so connected and their metrics are well measured. Unfortunately, as a legacy system, we do not have those numbers when it comes to the healthcare workforce”.

Interviewee #9 mentioned a strategy that is being developed and stated, “Yes, there is currently one that is being developed and run and we are part of it. So, there is work that is being coordinated by the Vision Realization Office with the involvement of the states, Ministry of Health, health loading company, Public Health Accreditation Board, and Saudi Commission for health specialties. So, these different entities have co-developed a program or plan strategy that was recently discussed with details”. Similarly, Interviewee #1 said, “There is a strategy of the national center and we are still working on developing it and it will see the light soon”.

#### 3.4.3. Areas of Planning That Require Exploration Sub-Theme

With regard to areas of planning that need more exploration, two of the study participants said that everything needs exploration with particular emphasis on specific areas. To illustrate, Interviewee #10 said that “all areas that lack Saudis, the turnover that happens, the fatigue element that happens with healthcare workers, and the psychological safety and comfort of the staff. However, I think all areas of all disciplines need proper workforce planning because I do not think we do it well”. Likewise, Interviewee #3 said, “Everything is important, but there is one area that has not been on our radar is the non-clinicians area”. Additionally, Interviewee #1 talked about three points and said, “First is the pure need based on the population. Second is the need based on the model of care. And the third thing is the Saudization of firms. All of these things are huge players in the scheme, but you have to start somewhere”. He then emphasized the need based on the model of care with an example, saying, “According to the World Health Organization (WHO), I need 500 vascular surgeons or one surgeon for every 10,000 population, but what are the roles of the vascular surgeon and what are the roles that someone else can do it on his/her behalf to allow him/her more time and space to cover more patients, so instead of one surgeon per 10,000, it can improve to become one per 100,000. What will the diabetic educator do, what will the prosthetics do, what will the health education and promotion worker do, and what will the health coach do for cholesterol, smoking cessation, weight loss reduction & blood sugar control?”

On the other hand, Interviewee #8 talked about the main goal of the National Center for Health Workforce Planning and said, “The most important thing is to make sure we do not have fragmentation of planning of the healthcare workforce, and that is the main goal of the National Center for Health Workforce Planning”. Interviewee #4 spoke with regard to numbers as an area that needs more exploration and said, “We will be much better off if we get real-time numbers about the people, workforce, and their jobs and positions,” and he explained with this example: “We did a small study on nurses in a particular sector and we got some conflicting reports; one set of experts said that we have an excess of staff in that particular unit, and other experts said that no we have a shortage. When we did a deep dive, we found that yes there is an excess of nursing staff in that unit but because many of them are doing administrative jobs and not taking care of patients, there was a shortage”. Lastly, Interviewee #7 answered that nursing needs more exploration.

#### 3.4.4. The Golden Rule for Workforce Planning Sub-Theme

While one interviewee answered that they do not have a golden rule after asking about their own golden rule for workforce planning, the others raised several ones. Interviewee #9 and Interviewee #7 emphasized data and metrics, saying, respectively, “One rule that needs actual attention is that whatever effort that is going to be put in workforce planning, it needs to be data-driven,” and “You need to have real-time knowledge of people and what they are doing; it is just on metrics. Once you know them, you know the demands and you can plan accordingly”. Additionally, Interviewee #3 said, “It is to have a golden goal, the proper definition for every word that you use, and then you need to define your scope of work; how horizontally broad and vertically deep”. On the other hand, Interviewee #6 said that “Workforce planning must be in a strong regulatory body and empowered by law strongly to have good direction and outcome to impose their planning on the health sector. I think they must be empowered”.

#### 3.4.5. Priorities That Determine the Needs to Meet the Vision for 2030 Sub-Theme

Regarding this, Interviewee #1, said that the needs are already clear, “We already know the needs, the model of care is clear, the need per population is clear, the job function needs more details to know how much you want, but at least there is an area that we can start with, and also the disease demographic is clear”. Otherwise, all other participants replied with different priorities. We can see this in Interviewee #2’s answer, as he responded with three priorities, saying, “First is workforce distribution because gaps and variations now are very high. Second, the specialties that have a high expert percentage, so need to be addressed. The third is closing the gap between Saudization and expatriates which will take a longer time to do”.

Meanwhile, Interviewee #10 mentioned workforce as a priority and said, “I think we should understand our current workforce as it is on the ground because I think there is a variation when it comes to different hospitals and organizations; it is not the same. We also need to look at how that compares to benchmarks, so for example ratios of nurses to the number of beds, are we looking at it in the same way? and how would it be at the rehab vs ICU vs other areas?” Similar to Interviewee #10, added one more, and said, “I think the priority is a proper plan for the workforce, have a proper plan for how you would like to see healthcare be delivered and the second is the education and training to make it happen”.

In terms of the demand and supply gap, Interviewee #4 said, “The priority is to ensure that the demand and supply gap is met. As we look at achieving the goals of 2030, we would want to ensure that the workforce that is needed is continuously supplied, to keep the system running”. Meanwhile, Interviewee #5 mentioned a solution for the latter issue, “The goals of the health transformation are the drivers because they were identified as the areas of pain; that we have to address. Therefore, I think all the initiatives and demands will lie there”.

On the other hand, Interviewee #8 spoke regarding a potentially vicious cycle, “The priorities should be, in addition to those who would come out of the needs assessment, utilizing the healthcare facilities, that is why we do not have adequate post-graduate training. If we do not have that adequacy, we will be in a vicious circle”. Lastly, Interviewee #3 listed some priorities: “Health, population growth, population transition/transmigration across the Kingdom, and socio-economic and political determinants”.

#### 3.4.6. The Need for a Change Management Strategy Sub-Theme

Study participants were asked around the way they anticipate the need for a clear change management strategy to enable health transformation. According to Interviewee #1, planning does not include change management initiatives; instead, it requires collaboration between multiple sectors, “You will have to get the right answer from the right person and it requires everyone to collaborate for the same vision,” he said. Conversely, Interviewee #9 assures that “There is a need, there is a dire need for a change management strategy implementation now at this stage”. Interviewee #6 agreed that change management is a strong strategy; however, he mentioned that “Change management is focusing on human factors. The problem that we are facing now is the human factor. When you neglect it, how would you build a good person, good healthcare professional to provide good quality and good care to people? When you do nothing, you will face that result”. Moreover, Interviewee #7 claimed that health transformation is a complex change, and change management is an integral part of this. 

Meanwhile, Interviewee #8 spoke about ways to achieve change, saying, “With change comes certain concerns and dynamics, and rather than being reactive to it, you should be proactive, and anticipate that. You have to include two main stakeholders; the providers, who are the front-line clinicians, and the recipients, who are the target population”. Additionally, Interviewee #4 discussed that an important aspect of achieving the vision is changing the minds of people, and said, “Changing the mindset of people is as important as doing anything else”.

#### 3.4.7. Workforce Upskilling Sub-Theme

Most of the study participants agreed that workforce upskilling is required to enable health transformation. On the contrary, Interviewee #1 suggested that reskilling is more important, where he mentioned, “Reskilling is basically when a person is not doing something and you train them to do something useful”. Instead, Interviewee #2 recommended continuous improvement and maintenance programs. Moreover, Interviewee #8 had a similar opinion on the importance of continuous quality improvement and upskilling. She mentioned that “Managers need to improve their skills and clinicians need to keep up with the changing developments in the field”.

On the other hand, Interviewee #5 stated that “It depends on what areas we are talking about, and it has to be based on an analysis that guides these initiatives”. Additionally, Interviewee #4 suggested something bigger than just continuous professional learning, saying, “We want the healthcare systems to be learning healthcare systems where every day you learn without having to look at it as a new skill; I would call it constant engagement in one’s task and profession, and it is required”.

#### 3.4.8. Attractive Health Ecosystem to the Workforce Sub-Theme

Interviewees were asked about how to make the health ecosystem more attractive for the health workforce. Interviewee #2 mentioned when he took nursing and the cultural stigma associated with it as an example: “Media campaigns that focus on the value and picture of nursing might give some good results”. Meanwhile, Interviewee #9 claimed that “I think the right incentives, financial and non-financial, are going to be needed”. Additionally, Interviewee #10 focused on promoting both extrinsic and intrinsic incentives. He stated, “Respect, being in an appreciative environment, understanding, allowing people to have courage and advocate for their colleagues and also understanding the fatigue element that comes with healthcare”. 

Another opinion shared was by Interviewee #5, where he said, “Making sure that we are addressing the healthcare system needs in terms of coordination of services, support to the healthcare practitioner team, or through a system that can be automated solutions or coordination of care between providers”. Lastly, Interviewee #1 discussed that there is a struggle with the various factors to achieve an attractive ecosystem, and he mentioned that programs like Daem have already been implemented to help with stress, burnout, and some academic difficulties: “Now we have the Daem framework that we give to people so they can implement Daem in their hospital”.

On the other hand, Interviewee #3 had a different perspective, wherein he expressed, “One of our big challenges is that the duties and responsibilities are not very clear. It is a combination of well-being, the environment, and fairness in contracting”. Moreover, Interviewee #7 went on to mention three things essential for an attractive health ecosystem—proper payment, benefits, work environment protection—and lastly proper training and education for healthcare workers, as he explained, “Healthcare workforce is badly underpaid. How would you convince those first of all to go to that healthcare industry and to stay there for the burnout they get?” On the other hand, Interviewee #8 focused on improving clinical units and housing conditions for people coming from outside, like nurses. Interviewee #4 claimed, “People are more incentivized to do things which have a purpose, in which they can attain mastery and in which they have autonomy”. Then, he discussed again the point he raised during the previous sub-theme, workforce upskilling, “All this is possible when the healthcare system becomes a learning healthcare system,” he said.

### 3.5. Healthcare Quality Theme

Regarding our last theme, healthcare quality, it combines the participants’ perception of their ideal picture of healthcare quality, current satisfaction of healthcare quality, how to enable the workforce with the enhancement of quality to meet the vision for 2030, and also their attitude towards technology concerning the quality of healthcare. Consequently, we have divided this theme into four sub-themes.

#### 3.5.1. Ideal Quality of Healthcare Sub-Theme

Interviewees were asked about their perceived notion of the ideal quality of healthcare. Interviewee #1 mentioned that the vision and mission of the Saudi Commission for Health Specialties is the definition of the ideal quality of healthcare, and he defined the ideal quality of healthcare as “A healthy society through competent healthcare professionals led by qualified health practitioners who serve the community with humanity and efficiency”. Additionally, Interviewee #11 claimed that the quality of healthcare provided here in Saudi Arabia is considered within the best, but he also added that “To be ideal, we need to keep up with the same scale and improvement”. Similarly, Interviewee #7 stated that “One of the most prevailed pride flags in Saudi Arabia is the healthcare industry. Healthcare in Saudi Arabia is very high quality, not only regional but also international”.

On the other hand, although Interviewee #5 mentioned that all systems are not perfect, he proceeded to list certain features of the ideal healthcare system, and said, “The system that is not soiled, addresses health before illness, engages with the public in their health, focused on value rather than process, and equitable to all people who are eligible rather than allowing people to go and hunt for their care”. The standards of the WHO, which is “to have a safe, efficient, effective, patient-centered, timely and equitable healthcare system,” were taken as a reference by Interviewee #10, in which he emphasized that complying with them will result in better healthcare quality. Similarly, Interviewee #8 stated, “There is a big room for improvement, we have the mechanism to help us at least now where we are and how we can move forward”. A different opinion was given by Interviewee #4 as he explained, “Quality is a comparative thing. It is something to constantly strive for and improve forever”.

#### 3.5.2. The Current State of Satisfaction of Healthcare Quality Sub-Theme

Most of the participants agreed that the quality of healthcare can always be improved from better to best due to the reasons that “Human life is precious so we should never be satisfied with any quality measure that we have” and “Quality is a continuous process,” as mentioned by Interviewee #1 and Interviewee #3, respectively. Moreover, Interviewee #2 described how difficult it is to determine, but emphasized on a general opinion, and said, “It is different from city to city and from sector to sector. There is still a lot of work to be done in the quality domain”. Similarly, others, particularly Interviewee #5, Interviewee #9, and Interviewee #10, also shared a similar opinion regarding the variability in the quality. To clarify, Interviewee #9 proceeded to say, “There is a big variation, not only across the healthcare institutions but also within the healthcare institution”.

Interviewee #10 added that “We do not have good regulations when it comes to quality measures and outcomes. I think if we were going to measure the actual quality of our healthcare system, we would be very surprised, and not in a good way”. Lastly, Interviewee #6 pointed out that “There is a problem with the accessibility to care, there are some shortages in some specialties and regions. So, we have a real problem that needs to be fixed”.

#### 3.5.3. Engaging the Workforce with the Enhancement of Quality to Meet the Vision for 2030 Sub-Theme

Study participants were asked about how they can engage the workforce to enhance the quality to meet the vision for 2030. A variety of opinions emerged. First, Interviewee #1 said that the workforce is already engaged, as he said, “I do not think anyone will refuse to improve patient outcomes; it is inhumane and impossible. The healthcare practitioners, by definition, to live properly, have to have something called reflective practice”. Moreover, Interviewee #9 emphasized that “It is important they understand there is a meaning of what they are doing, that they are engaged in the change that is happening, and they are not excluded. You need to power the clinicians and clinical leadership. In terms of clinicians, they need to be an essential part of the main driver of the change along with the support of rulers”. Likewise, Interviewee #5 mentioned engaging healthcare workers in the design process because, eventually, they are the ones who are going to implement it. Similarly, Interviewee #8 talked about coproduction and the importance of representing and involving healthcare practitioners. Interviewee #2 argued with regard to the probable current statistics with the emphasis on performing better, saying, “We might find less than 10% who are engaged. So, we need to bring people aboard, convince them of the vision, communicate the strategy to them, engage them in projects that will benefit the strategy, and put in an incentive system to reward them”.

On the other hand, Interviewee #10 spoke about promoting patient centricity in everything that is done, stating, “If you live your values rather than them just being words on a banner, you are going to get the staff and the belief system”. Meanwhile, Interviewee #6 mentioned the current plans and said, “We have comprehensive plans with regards to the workforce. Creating governance to the undergraduates in education in some specialties as a suggestion to have a relationship to enhance the education as an alignment between the Ministry of Education and Ministry of Health to achieve the needs of the healthcare sector”. However, Interviewee #3 mentioned five strategies, which are teaching quality from early training days, transparency, continuous training, giving them numbers, and, lastly, incentives in return to good quality care.

#### 3.5.4. The Importance of Technology Sub-Theme

Most of the interviewees agreed that technology is a substantial part of the transformation. First, the statement of the use of applications during the COVID pandemic as successful use of technology was mentioned by Interviewee #2. Meanwhile, Interviewee #3 mentioned the role of technology in decreasing human error but relying on automated decisions. According to Dr. Saleh, technology can play a big role in measuring things which in turn affects the quality and delivery of healthcare. “Most important thing to do is to measure things. Quality is nothing but measuring things,” he said. Interviewee #6 stated the importance of the use of artificial intelligence in the predictability of the workforce and the market. On the contrary, Interviewee #10 mentioned technology as a double-edged sword: “You can either use it very well and it can enhance the work that you can do, especially things like AI, predictive AI, looking or reading radiology or lab results. At the same time, if we rely too much on technology and remove the human element, we are taking away part of the empathy that comes with healthcare and I think we already do that with all the documentation that we do”.

## 4. Discussion

As the quality and further enhancement of the healthcare workforce are considered crucial parts of the Kingdom’s 2030 Vision and to the great exposure and experience of healthcare leaders toward workforce planning, this research aimed to investigate the attitudes and perceptions of the healthcare leaders towards quality enhancement of the healthcare workforce, and therefore understand the current status of the healthcare workforce. Through the use of a semi-structured interview, we were able to understand the attitudes and perceptions of each healthcare leader that participated in the study with regard to workforce quality enhancement. The results provided different opinions, including positives and negatives, several suggestions to various areas, and also the current efforts. 

### 4.1. Workforce Competency Theme

#### 4.1.1. Current Status of the Workforce Competency Sub-Theme

Healthcare workforce competency is an area that one can never be satisfied with. Although most of the study participants were happy with Saudi Arabia’s progress over the previous years in terms of competency, there is always room for improvement. With Saudi Arabia’s Vision for 2030, the new model of care (MOC) is centered around physical, mental, and social well-being, with the patient outcome being the main focus. To fulfill the vision, achieve new goals, and modernize the healthcare sector, it is necessary to incorporate new strategies [12]. Competency-based medical education (CBME) is an approach that integrates competency into education early on and will eventually aid in fulfilling the needs of the twenty-first century. Moreover, deliberate practice is a helpful strategy in improving competency, where learners commence to refine their practice with constant reflection, feedback, and practice until mastery is achieved [13]. 

Attention must also be given to allied health and nursing. A study by Feliciano et al. mentioned that Saudi Arabia is still facing a shortage of nurses, where 63.82% of the nurses working in the Kingdom are expatriates. They also showed that various factors can affect nurse competencies, including duty hours, nurse–patient ratio, marital status, years of graduation, and length of service [3]. In addition, apart from improving competency, unifying it across all hospitals and areas of the Kingdom is important. Currently, competency can be variable depending on the specialties and geographical locations. A study in Australia showed the presence of workforce maldistribution, with regard to both geographical location and skill mix [14]. Rural areas face significant problems regarding the accessibility, availability, and appropriateness of health services [14].

#### 4.1.2. Barriers of Capability Building Sub-Theme

When we talk about the barriers that are hindering the fulfillment of the vision with regard to facing capability building of the workforce, serious barriers were mentioned in three aspects. The first barrier that has emerged is the fragmented system because we have eight independent healthcare providers also with the private sector, and it was pointed out that it is considered the main barrier. In addition, a solution for this fragmentation was proposed, which is to take education and post-graduate training that are unified as an opportunity to make the system completely unified by making the proper analysis and diagnostics to treat the issues in the private and public sectors and charities. A study conducted by the European Observatory on Health Systems and Policies mentioned that improving coordination inside and across levels of care would enhance efficiency even further [15]. One of the complexities of the system is that we are facing a workforce that comes from different backgrounds and works in different places [15]. 

The second barrier that was noted is the utilization of services. To illustrate, mis-utilized services may have an indirect impact on training since there will be a lack of cases. As poor access to healthcare services is considered one of the critical aspects for the utilization of the system, a study found that poor access to healthcare services is likely to lead to low numbers of cases, thus restricting training [16]. 

The third barrier is the uncertainty of the future and the talent pool. The unpredictability of the future is a big obstacle to creating labor capacity, and you cannot build capacity based on exigencies, but rather on what is predicted on the path of growth. There is also a workforce barrier in terms of the talent pool, which we must leverage in all elements of the workforce to develop them. Finally, we have constraints in work opportunities and the entering pool, and the proposed solution is to have companies that are ready to take entry-level competence. However, no research was found to support nor argue against this concept. 

#### 4.1.3. Enhancement of the Workforce Competency Sub-Theme

The current enhancements that are taking place are within the health academy and leadership academy. The yealth academy is directed to address the workforce skill gap, and it was pointed out that continuous improvement of this task using numbers to measure the adequacy of these initiatives is essential. However, the leadership academy is present to handle the administrative skill gap, aiming to create opportunities for such training, and to create frameworks, admin programs, leadership journeys, and digital platforms for self-training. In 2019, a study was done in Saudi Arabia, titled “Building the health workforce: Saudi Arabia’s challenges in achieving Vision 2030” [17]. This study has found various areas that need proper planning and strategies, one of which is skill gaps [17]. Thus, building a health academy for the workforce, to aid in filling the gaps of workforce skills, is crucial at this point.

Another current movement that is being undertaken by the SCFHS is including more hospitals to be part of the post-graduate training, and qualifying and providing more trainers within these hospitals. A similar point was raised, but it was rather a suggestion for a better engagement of undergraduate and postgraduate education and training. For the training to be more beneficial, comprehensive data analysis should run from all vast healthcare and education, including universities, SCFHS, and hospital training. In addition, a study that was conducted in India in 2020 stated that the training of healthcare workers has been identified as one of the important strategies under the National Patient Safety Implementation Framework (NPSIF) as well as in the WHO’s regional strategy [1]. 

On the other hand, different ideas were raised. Two topics were suggested and they combined one concept, which is consistency. First, recertification was claimed as one of the things that will guarantee consistent competency. Second is the intervention for enhancement, where checking is based on three interference points: first is at the point of entry, second is after graduation, and third is constant learning. As explained in a study, competency is considered a product of an extending cycle, from the initial phase, developing knowledge and skills, to application, and then repetitive reflections and feedback to ensure consistency and further enhancements [18]. 

Moreover, testing students even before graduation from their colleges by applying universal standards using competency assessment tools by the SCFHS was suggested. The perspective of this point was due to the reason that graduating from a Saudi university yields vast and different outputs. A further suggestion was to tap into scientific committees as well as scientific bodies that are independent and may help develop competency assessment tools. Upon further literature review, no study was found to be discussing this particular suggestion.

### 4.2. Health Transformation

#### 4.2.1. Components of the Health Transformation Strategy Sub-Theme

The Health Transformation strategy was initiated back in 2016 as a small part of the life quality stream, and it is now a major part of the 2030 vision, and an independent strategy, called the Health Transformation strategy. Even though the progress of the health system is indeed prominent in Saudi Arabia in the past decades, the development of the system is mandatory for the achievement of the 2030 vision of the Kingdom, as stated in a previous study [12]. 

However, three critical points ought to be met for the health transformation to be successful. The first point comes from an economical perspective where the outcome must meet the governmental spending. Second is the regular accessibility to the healthcare system by moving from the model that is based on the hospitals and institutions to more around the center. Lastly, improvement of healthcare services in all regions is the third, focusing on reducing the burden of chronic diseases and reducing the impact of accidental injuries. Regarding accessibility, a study conducted in Australia in 2021 by Guillam et al. showed that there is an association between limited healthcare services access staffed by qualified physicians and poor health outcomes, which includes a decrease in cancer survival rates and increased complications of diabetes [14]. 

The challenge of the Health Transformation strategy is that the system cannot stop implementing the transformation planning while still aspiring to provide the best services with the highest value in the least possible time. The population of the Kingdom persists on growing and it is anticipated to be 39.5 million, with 4.63 million elderly by 2030 [12]. Moreover, the rates of preventable accidents and non-communicable diseases are considered high in the Kingdom when compared to regional and international standards [12]. With regard to where we are currently, the new MOC has been unfolded as mentioned before and the Public Health Program (PHP), which is a new system now where it purchases and funds healthcare services. 

#### 4.2.2. Concept of Value-Based Healthcare Sub-Theme

Value-based healthcare is one of the important objectives of the health transformation strategy in the Kingdom of Saudi Arabia’s vision for 2030. The definition of it is minimizing cost and maximizing the value, and that is the primary motivator. Moreover, it is aimed to achieve the concept of building the fund for the outcomes instead of funding for the inputs or tools. Moreover, it is meant to be the future of healthcare quality and services that are given to the patient and the system as previously mentioned. In another unique perspective, value-based healthcare was determined by those who assess the value, and the main assessment is done by the patient and the nation. A study conducted in 2020 found that achieving the real meaning of value-based healthcare (which is that the needs of each patient are individualized, and it requires continuous improvement from an interdisciplinary team approach) mandates incorporation of the principles and implementation of value-based healthcare in the undergraduate curriculums to prepare the next generations for the proper execution of value-based healthcare [19].

#### 4.2.3. New Roles in Healthcare Transformation Strategy Sub-Theme

By 2030, it is aspired to reach an average life expectancy from 74 to 80, and it is an ambitious goal. Therefore, not only do we need to increase the existing specialties, but we also have to be thinking of new ones. Thus, the National Healthcare Center of Workforce for Planning is an important catalyst for the cause of this transformation, and implementing new roles is one of the center’s tasks. Although the importance of having new jobs and skills have been demonstrated, carrying out proper analyses and studies originating from our environment to find the specific needed roles is just as important. Now, the health academy has assigned 29 new roles for the transformation strategy. A study showed the introduction of new roles into an already built system requires planning and conduction, and mainly focuses on patients’ needs and a well-defined scope of practice [20]. 

As for the roles that were demonstrated in this study, they are AI specialists, coders, case managers, coordinators, health coaches, genomicists, health economists, multilinguals, and politicians. However, AI is used sporadically in the current presence in the form of what is called, “artificial narrow intelligence”. In a study where the impact on the adoption of AI was investigated, Krishnamoorthy et al. suggested that the only dimension that appeared to be impacting the adoption of AI is the high uncertainty avoidance [21]. Moreover, there is a dire need for coders since the number of trainees produced annually does not meet the need. Nevertheless, reprioritization of the current jobs and the need for enhanced leadership training rather than new titles have been claimed. Previous research which was focusing on nursing found that developing transformational and relational leadership could reinforce nurse satisfaction, recruitment, retention, and a healthy work environment [22]. 

#### 4.2.4. Incorporation of Non-Clinicians as Part of the Healthcare Workforce Sub-Theme

We have non-clinicians working in the healthcare system, but they must be prepared via adequate education and training, which is being accomplished. Non-clinical interactions play an important role in preventive healthcare; consequently, everything linked to preventive healthcare could be non-clinical. On the contrary, various sectors require non-clinical services and expertise; it will address the need for costing, coding, data, and information, and these are the primarily non-clinical responsibilities that will necessitate significant effort to narrow the gap. They also provide enablers for healthcare services such as financial, legal, human resources, and people administration. As a result, we need to extend our workforce strategy as an infrastructure workforce to provide high-level healthcare services. According to one research, non-clinical personnel play an important role in a patient’s healthcare experiences since they are often a patient’s first point of contact with health services [23]. 

#### 4.2.5. Privatization and Saudization in Healthcare Transformation Strategy Sub-Theme

First of all, privatization is being implemented in a corporatization manner where the clusters are being privatized. Additionally, they are not owned by anyone except the government, and that is called “institutional transformation”. From the view of the private sectors, however, the purpose of privatization is not for increasing the margin of interest, it is rather to forcefully engage the private sectors in increasing GDP and partnering with the government. Moreover, it helps with efficiency and productivity by creating value, transparency, and accountability, and by closing the workforce gap. The main purpose of privatization is to have a flexible system focusing more on outcomes and performance where incentives are created for rewarding and promoting and to know the value chain of money flow from the first minute to the last. Meanwhile, the worst issue that could be faced is when privatization is looked at as a must to achieve, as pointed out. Furthermore, the civil service system is a huge obstacle to the enhancement of the health workforce. A study showed previously that the answers to privatization and corporatization questions are not clear, and the evidence is little and mixed [24]. 

Subsequently, Saudization is considered an ultimate goal and a strategic objective for the country, and its goal is to add as many Saudis as possible in the system, not 100% Saudis while focusing on delivering value-based healthcare services. In addition, there is a plan to have a minimum standard of accepting non-Saudis in certain areas. The believed reasons behind this movement are that it can create stability in the workforce, more satisfaction from the population, security, and to benefit from local content and talent. In addition, employing non-Saudis has its benefits, which are enriching the experience and to maintain competition between Saudis and non-Saudis. Yet, Saudization has to be thought about in a longer-term plan and invested in. Nevertheless, the shortage of trained healthcare physicians with the huge dependency on expatriates is of great barrier, but the implementation of Saudization has seen considerable success in raising the targets in the private sector over the past years [25]. 

### 4.3. Leadership

#### 4.3.1. The Current Healthcare Leadership Qualities Sub-Theme

Feedbacks on the current status of healthcare leadership qualities were mixed, yet it was mostly negative. Leadership in the healthcare sector needs distributive leadership in the sense that any decision-maker is a leader, whatever level they are at. Now, mostly identified the issue with leadership as if the employment is based on seniority or clinical knowledge rather than leadership skills. Jeyaraman et al. showed various returns on investment in healthcare leadership development programs; most prevalent are increased patient satisfaction, increased job satisfaction, better patient care quality, decreased burn-out, fewer patient complaints, and greater leadership competencies/skills [26]. 

Furthermore, investing in the younger population might allow the discovery of emerging and future leaders of the organizations. In previous research by the National Association of Colleges and Employers, it was found that more than 80% of employers require “leadership” in applicants’ resumes [27]. Moreover, leadership needs to be developed into the new concept of a learning organization and adaptive leadership. Since organizations are in continuous change, adaptive leadership is beneficial, and it is called transformational leadership, where it encourages followers by captivating them to better ideas and moral values [28]. 

#### 4.3.2. Areas of Enhancement in the Criteria of Leader Employment Sub-Theme

It is critical to invest in leaders, those who operate as part of a team and grasp the overall goal. There is a lack of effective planning and execution, and additional education and training. Besides this, supervision for a certain period can improve these areas. The key to leadership is that they must be daring, open-minded, have social skills, team building, communication skills, and a sense of justice to work with one another since they will not succeed alone. A study showed that people-centered leadership strategies help to improve results for the nursing workforce [19]. Most healthcare practices require some level of leadership, starting with graduate training will play a significant role in implementing leadership characteristics. So that is where we should start. 

Now, the SCFHS has done several examinations of acquired skills within the healthcare system. There is currently a leadership academy within the SCFHS, and it created a model with two core competencies or values: accountability and caring. Additionally, five skills and eighteen behaviors should be nurtured in leaders based on several workshops with Saudi healthcare experts after the revision of organizational psychologists. As a result, every program that was developed has been built on this model.

### 4.4. Workforce Planning

#### 4.4.1. Importance of Planning Strategies Sub-Theme

Healthcare workforce planning aims to provide the ideal workforce to ensure optimum patient care in terms of quality, timing, place, skills, and even cost [29]. With increasing future healthcare demands and the adoption of health transformation strategies, the importance of proper workforce planning increases despite its complexity. With clear long-term goals to achieve, planning starts by comparing current capabilities with future needs, after which, one can map resource requirements based on staffing levels, skill sets, locations, and hiring costs [2]. 

However, faulty planning can have a negative impact including a workforce saturated in certain fields or regions while lacking in others, leading to a lack of utilization of valuable skills and knowledge. According to Pastores et al., in the United States by 2030, the nursing shortage could exceed one million [5]. Some fields rely heavily on the foreign workforce; thereby, it is wise to include strategies of recruitment of international workforce and grooming the local workforce to be able to decrease reliance on expatriates. Saudi nationals constitute 44% of all health workforce and 29.5% of physicians [1]. For efficiency and productivity, it is important to also consider the tools and variables used to set up planning strategies. Outdated, unspecific, and unreliable tools can negatively impact the plan and its implementation. 

#### 4.4.2. Examples of Adopted Strategies Sub-Theme

Workforce planning is both a science (analysis) and an art (execution) [2]. One of the strategies mentioned was focusing on the fragmentation issue in healthcare. Plans, priorities, and goals of various healthcare sectors should ultimately unite to serve the national vision of optimal patient-centered care. Being interconnected and having data accessible from various sectors makes planning and execution more realistic adding fluidity and ultimately better outcomes. In a previous study, Health Workforce Australia (HWA) implemented main principles to warrant success in national health workforce planning [30]. They include updating as new data appears, periodical revising, and improving data collection [30]. 

At present, there is coordination between the Vision Realization Office, Ministry of Health, health loading company, and Public Health Accreditation Board, and the SCFHS has been established to co-develop a program or plan strategy for the Health Transformation Vision 2030. Moreover, there are comprehensive plans to create governance for undergraduate education as an alignment between the Ministry of Education and Ministry of Health to enhance education and achieve the needs of the healthcare sector. 

#### 4.4.3. Areas of Planning That Require Exploration Sub-Theme

Proper planning includes reflection and exploration. Although exploration is required in all aspects including psychological safety of healthcare workers, turnover, fatigue element, and comfort of staff, a greater emphasis should be given to non-clinicians. A study done in the United States showed that clinicians were 1.45 to 1.78 times more likely than non-clinicians to recognize the importance of their role in disaster response [31]. In addition, an emphasis on exploring and delineating the description of each healthcare worker must be given to ensure optimal distribution of efforts and skills. Decreasing fragmentation between healthcare sectors and working on getting real-time data are other areas requiring exploration.

#### 4.4.4. Golden Rule for Workforce Planning Sub-Theme

One of the main rules mentioned was the effective utilization of real-time data in planning. Defining the terminologies and the scope of work is also important.

#### 4.4.5. Priorities That Determine the Needs to Meet the Vision for 2030 Sub-Theme

With the establishment of the healthcare transformation strategy, the needs are clear to meet the goal. Priorities include appropriate workforce distribution, decreasing the percentage of expatriates in certain specialties, and closing the gap between Saudization and expatriates. Attention must be given to the current status and the existence of any variability in comparison to benchmarks. Albejaidi et al. stated that the elderly population is projected to increase from 3% in 2010 to 18.4% by 2035 [1]. The study also showed that the number of physicians per 10,000 population was 9.4 during 2005–2012 and increased to 24.9 during 2007–2013 [1]. The demand and supply gap should be met.

#### 4.4.6. The Need for a Change Management Strategy Sub-Theme

Change management according to John Kotter has emotional and situational perspectives and occurs in eight steps: developing urgency, building a guiding team, creating a vision, communicating for buy-in, enabling action, creating short-term wins, do not let up, and making it stick [32]. On the other hand, William Bridges relates change with the change of identity happening in three steps: endings, the neutral zone, and beginnings [32]. Planning initially requires collaboration between multiple sectors then change management comes at a later stage as an integral part of health transformation. It is important to be proactive and anticipate change rather than being reactive to it.

#### 4.4.7. Workforce Upskilling Sub-Theme

Continuous quality improvement and workforce upskilling are important to keep up with the vision for 2030 goals. Reskilling, in which a person is trained to do something useful, is also crucial to utilize the workforce skills and capabilities. Ideally, healthcare must be a learning healthcare system where skills are gained with constant engagement in one’s duties. A study in 2021 showed that the new European Skills Agenda is aiming at upskilling and reskilling in the next five years [33]. Moreover, the new skills should be included in the curricula of medical colleges and incorporated into continuous professional development (CPD) [33]. 

#### 4.4.8. Attractive Health Ecosystem to the Workforce Sub-Theme

The health ecosystem is important to the healthcare worker’s ability to provide optimal patient care. Appropriate financial and non-financial incentives can greatly impact the health ecosystem. Incentives include respect, an appreciative environment, courage for advocacy, and understanding fatigue. Coordination and use of automated services between providers make services faster and easier. Clarifying the duties and responsibilities of healthcare workers in addition to proper payments, work environment protection, and proper training and education are also essential for a better healthcare ecosystem. The foreign workforce must also be taken into consideration by providing them with suitable clinical units and housing conditions. As shown in a previous study, the high workload may hurt practitioners’ wellness, team perception of care quality, time available for teaching, and length of stay [5]. Currently, there exists a program that deals with stress, burnout, and academic difficulties, called Daem.

### 4.5. Healthcare Quality

#### 4.5.1. Ideal Quality of Healthcare Sub-Theme

A perfect system is impossible to apply. Yet, the perceived ideality of the quality of the healthcare system is when the system is striving for improvement constantly and comparatively. In prior research, quality improvement principles showed effects on improving quality and patient satisfaction [34]. One of the most prevailing pride flags in Saudi Arabia is the healthcare industry, and the vision and mission of the SCFHS are for the idealization of the quality of healthcare. In 2016, a systematic review in Saudi Arabia found that there is big room for quality improvement in university hospitals [35]. Yet, no novel article has been found discussing the quality of healthcare in Saudi Arabia. 

#### 4.5.2. Current State of Satisfaction of Healthcare Quality

As discussed previously, the quality of healthcare in Saudi Arabia is generally decent. However, the current satisfaction and attitudes are complex. Indeed, satisfaction is a continuous process and can never reach climax since it correlates with precious human life. Aside from the general quality of healthcare, we face great variations, not only across the healthcare but within a healthcare institution as well. Since accessibility is an integral part of health transformation strategies, the present satisfaction of this particular area is unfavorable. 

#### 4.5.3. Engaging the Workforce with the Enhancement of Quality to Meet the Vision for 2030

The workforce is already engaged to improve quality in the form of reflective practice. As stated in an article, reflective practice is aligned with career progression in healthcare [36,37]. Indeed, clinicians, with the support of leaders, are the leading driver of change. In case of further engagement, teaching quality from early training days, transparency, continuous training, communicating the strategy and vision, further engaging in projects, and putting an incentive system for rewards are potential strategies. 

#### 4.5.4. The importance of Technology

Technology is an integral part of the transformation. Since quality is dependent on measurements, technology benefits it substantially, also in the delivery of healthcare. Additionally, it helps in the predictability of the workforce and market when AI is used correctly. 

However, technology can be a double-edged sword: when used correctly, it can enhance the workforce, yet it could remove the human element, thus taking away empathy. Moreover, empathy is currently affected by the huge amount of documentation that is present. A systemic review showed there is a need for solid research on the risks of utilizing technologies and their cost-effectiveness [38]. 

In Table 3, below, we provide a summary of the key findings of our research.

## 5. Conclusions

Our research study tried to understand the Attitudes and Perceptions of Health Leaders for the Quality Enhancement of Workforce in Saudi Arabia. Our ambitious objective was linked to a systematic analysis of 5 themes and 22 subthemes providing very important insights for the revision of the Workforce Planning Strategy. 

With the implementation of MOC and CBME, healthcare leaders in Saudi Arabia are satisfied with the direction of workforce competency. With the huge variability of competency resulting from the system fragmentation, it is substantial to unify all hospitals of the Kingdom. Moreover, healthcare leaders intensified the misutilization of services and the uncertainty of the future and talent pool as potential barriers for capability building. 

As health transformation is an integral part of the Kingdom’s vision for 2030, it has to be successful. However, three points must be met, which are value-based healthcare, regular accessibility, and continuous improvement of healthcare services. 

Currently, assigning new roles could be more important than the enhancement of workforce quality. Thus, the health academy in the National Healthcare Center of Workforce has determined 29 new roles for the transformation strategy. In addition, AI and coders were highlighted strongly in this study because of their insufficiencies and importance. 

There is a gap in the workforce; thus, implementing privatization might help with closing this gap, also with efficiency and productivity by creating value and with transparency and accountability. However, the execution of privatization is aimed to corporatize the private sector, and therefore, be owned by the government. Aside from privatization, there is a plan currently to have a minimum standard of accepting non-Saudis in certain areas to maintain the competition between Saudis and non-Saudis and to enrich experience while still achieving the Saudization goal. 

Now, there is coordination between the Vision Realization Office, Ministry of Health, health loading company, Public Health Accreditation Board, and SCFHS to co-develop a workforce plan for the Health Transformation Vision for 2030. In addition, one of the initiatives is to solve the fragmentation issue because it will aid with data accessibility and interconnection making planning and execution more realistic. Yet, for now, planning requires collaboration between multiple sectors.

There are high satisfactory attitudes towards the healthcare quality in Saudi Arabia from the healthcare leaders. However, accessibility and fragmentation are areas that need proper attention. 

## 6. Limitations

During the data collection, leaders were unavailable most of the time. Thus, a delay in the completion of the study occurred. Moreover, with the fast and ongoing health transformation in Saudi Arabia, research needs to catch up. 

## Figures and Tables

**Table 1:** Interviewees coding.

Participant	Hypothetical Name
Interviewee 1	Dr. Ahmed
Interviewee 2	Dr. Abdulaziz
Interviewee 3	Dr. Yazan
Interviewee 4	Dr. Saleh
Interviewee 5	Dr. Hamed
Interviewee 6	Dr. Bandar
Interviewee 7	Dr. Adnan
Interviewee 8	Dr. Fatima
Interviewee 9	Dr. Ali
Interviewee 10	Dr. Salem
Interviewee 11	Dr. Khalid

**Table 2:** Workforce Competency overview.

Areas	Key Facts	Interpretation
Strengths/Facts	- All our **education is competency-based** - Quality indicators in hospitals and our Saudization percentage is very high - **Qualifications and competencies** are measured and monitored with the best **standards**. Saudi Arabia now is one of the best five - The Kingdom has one of the **best workforces** in the world from the perspective of **quality and quantity**	- Competency-based Education - Continuous measurement of Quality / Adoption of international standards - The Saudi Workforce is one of the best in the World - Excellent perception of the status of health workforce
Areas for Improvement/Gaps	- I think workforce competency needs to be improved always, especially when we shift our paradigm from income to outcome. When we do this shift, we will have lenses that can show workforce competency - I think there is a gap even in the medical and surgical staff, but I think the gap is much higher in allied health and nursing with and an existing gap in the medical - The workforce should be viewed through the different clinical specialties”. Further, she stated that “When we are talking about the workforce we are talking about knowledge, skills, and attitude. It’s variable from one specialty to another and varies from hospitals and sectors. I would say there is a big room for improvement”. - Attracting the right people, bright talent is one issue or challenge, the other thing is providing a template for training, upgrading, and upskilling of the workforce to meet the required competency is another challenge. As well, given the transformation, there are various work roles that the market does not have enough workforce to meet these competencies and these needs	- Focused initiatives are required - Overall, very good perception of the current competency and efficiency of the workforce - Gaps exist in medical and surgical staff and also in allied health and nursing - Update on knowledge, skills, and attitude that varies from one specialty to the other and from one hospital to the other - Attracting the right people, bright people - Provide standardization and template for training - Upgrading and upskilling the workforce - The Transformation generates new workforce specialties opportunities (lack of personnel)
Barriers	- **The System** as one of the barriers. - The fragmentation of the system is the main barrier because we have 8 healthcare providers and the private sector, they are independent systems - The system is complex due to the workforce coming from different backgrounds and working in different places. The complexity of the healthcare system, in general, makes it quite challenging to work on the different levels required and different skills. - **The Services**, - The effect of unutilized services on training and responded with “The most important part is to have fully utilized services, but if they do not have the utilized services, they will not have enough cases and that would affect the training. So, this is the biggest barrier”. - **The uncertainty of the future**: “You can build capacity for what is an expected path of growth, you cannot build capacity for emergencies. - What we can do is we can **bring agility for emergencies, or resilience**”. - We have a workforce barrier in terms of the talent pool that we have to use in the workforce, and that fits into almost every other aspect of workforce competency”. - We have **limitations in job opportunities and the incoming pool**, offered a way out, - “We need to have employers that are willing to **accept entry-level expertise**”.	- The Fragmentation of the System - The Complexity of the System - The unutilized Services - The uncertainty of the future - The need to bring agility for emergencies or resilience - Limitations in job opportunities and the incoming pool
Challenges/Opportunities	- I think there are a lot of potential opportunities that are not being utilized properly. - We have **a unified system for education and postgraduate training**, so that is an opportunity like we are not a fragmented system as you will think in other countries and other opportunities that are not utilized properly. - Once you have a unified system, **make the proper analysis and diagnostics**, then you will be **working on a solution to treat these issues in the private sector, public sector, charities**, and all that. There will be some variations. I don’t see them as a challenge. I see the challenge is not doing what is supposed to be done	- The challenging unified system for education and postgraduate training is a great asset - Integrative approach for solutions that will involve the private sector, public sector, charities
Enhancement	- The current efforts that are taking place. - There is a current task that is being carried out, and, afterward, he pointed out an idea for the continuous improvement of the task - The Saudi Commission has a close relationship with this task through the expansion in between the programs that it imparts on in the past few years as a part of its strategic plan. - The establishment of the health academy that is directed to address this skill gap in the workforce - Now enhancement is on two levels, which are clinical and technical skills, and the efforts for these are minimal to moderate. However, admin skills are completely absent, so we are trying here in the leadership academy to create opportunities for this kind of training - Creating frameworks; admin programs; leadership journeys; and digital platforms so people can train themselves. - There is a lack of competency in the organizational structure which includes HR functions, talent management, succession planning, strategy implementation, and other things that should be in the healthcare system, and that is the reason for building workforce planning because it is needed to fill that gap. - We need to have very good training. So, we need to improve it in quantity and include more hospitals to be part of the postgraduate training, - We need to qualify more trainers within those hospitals, which is what the Saudi Commission of Healthcare Specialties is doing. - “I think we should run a comprehensive data analysis when it comes to competency and run this gap with all vast healthcare and education from universities, colleges, Saudi commission, and hospital training. - Once we do that gap analysis, from my perspective, I am sure that others will share their concerns and try to have a unified comprehensive solution that engages undergraduate and postgradu-ate education and training”. - Recertification is one of the things that would guarantee, after a longer period, that we still have the competency to practice. - Three points of intervention for enhancement, the first one is checking the workforce at the point of entry, the second one is after graduation, and the third is constant learning. - If you have well-known standardized competency, you can develop a road map towards closing these gaps that will influence your training programs, the resources that you need to close these gaps in terms of training requirements and others”. - I think we have to involve really good standards in place where we are testing our students even before they graduate from internships and not leave it to the hospitals where they train - Anyone who graduates should have a competency assessment by the Saudi Health Commission rather than relying on the fact that they just graduated from a Saudi university because the outputs of these universities are very vast and different and I think that we could always tap into scientific committees that exist and as well as scientific bodies that are independent that can help develop these competency assessments for the students or the applicants coming into the workforce and also ongoing assessment of competency.	- Bold current initiatives for enhancement are taking place - The implemented Strategy the last years in the SCFHS has a strong positive impact - The Health Academy is a key enabler of value delivery - Enhancement related to clinical and technical skills - Admin skills are absent and need enhancement - Special focus on creating frameworks; admin programs; leadership journeys; and digital platforms - Lack of competency in Organizational Structure (HR functions, talent management, succession planning, strategy implementation) - Focus on the Quality of Training - Focus on the availability of top-quality trainers - A comprehensive data analysis related to competency must run and to investigate the gap with all vast healthcare and education from universities, colleges, Saudi commission, and hospital training - This will lead to a comprehensive solution that engages undergraduate and postgraduate education and training - Recertification is a bold initiative to secure competency - Continuous assessment of workforce competency - Need for a standardized competency framework that will inform a roadmap for development - Competency assessment must be a continuous best practice

**Table 3:** Themes, sub-themes, and key findings.

Theme	Sub-Theme	Key Findings
Workforce Competency Theme	Status of the workforce	Very good assessment of the workforce competency
	Barriers of capability building of the workforce sub-theme	The complexity and the fragmentation of the systems The underutilized services
	Enhancement of the workforce competency sub-theme	Undergoing initiatives The implemented strategy in the last years in the SCFHS has a strong positive impact The Health Academy is a key enabler of value delivery Enhancement related to clinical and technical skills Admin skills are absent and need enhancement Lack of competency in Organizational Structure (HR functions, talent management, succession planning, strategy implementation) Focus on the Quality of Training Focus on the availability of top-quality trainers A comprehensive data gap analysis related to competency
Health Transformation Theme	Components of healthcare transformation sub-theme	Three critical points ought to be met for the health transformation to be successful: ▪ The first point comes from an economical perspective where the outcome must meet the governmental spending. ▪ Second is the regular accessibility to the healthcare system by moving from the model that is based on the hospitals and institutions to more around the center. ▪ Improvement of healthcare services in all regions is the third, focusing on reducing the burden of chronic diseases and reducing the impact of accidental injuries.
	Concept of value-based healthcare sub-theme	The definition of it is minimizing cost and maximizing the value, and that is the primary motivator. Additionally, it is aimed to achieve the concept of building the fund for the outcomes instead of funding for the inputs or tools. Moreover, it is meant to be the future of healthcare quality and services that are given to the patient and the system as previously mentioned. In another unique perspective, value-based healthcare was determined by those who assess the value, and the main assessment is done by the patient and the nation.
	New roles in healthcare transformation strategy sub-theme	Although the importance of having new jobs and skills have been demonstrated, carrying out proper analyses and studies originating from our environment to find the specific needed roles is just as important. AI specialists, coders, case managers, coordinators, health coaches, genomicists, health economists, multilinguals, and politists.
	Incorporation of non-clinicians as part of the healthcare workforce sub-theme	We have non-clinicians working in the healthcare system, but they must be prepared via adequate education and training, which is being accomplished. Non-clinical interactions play an important role in preventive healthcare; consequently, everything linked to preventive healthcare could be non-clinical. On the contrary, various sectors require non-clinical services and expertise; it will address the need for costing, coding, data, and information; these are the primarily non-clinical responsibilities that will necessitate significant effort to narrow the gap.
	Privatization and Saudization in healthcare transformation strategy sub-theme	Saudization is considered an ultimate goal and a strategic objective for the country, and its goal is to add as many Saudis as possible in the system, not 100% Saudis while focusing on delivering value-based healthcare services. Moreover, there is a plan to have a minimum standard of accepting non-Saudis in certain areas. Nevertheless, the shortage of trained healthcare physicians with the huge dependency on expatriates is of great barrier, but the implementation of Saudization has seen considerable success in raising the targets in the private sector over the past years
Leadership Theme	Current healthcare leadership qualities sub-theme	Feedbacks on the current status of healthcare leadership qualities were mixed, yet it was mostly negative. Now, most identified the issue with leadership as if the employment is based on seniority or clinical knowledge rather than leadership skills. Investing in the younger population might allow the discovery of emerging and future leaders of the organizations.
	Areas of enhancement in the criteria of leader employment sub-theme	The SCFHS has done several examinations of acquired skills within the healthcare system. There is currently a leadership academy within the SCFHS, and it created a model with two core competencies or values: accountability and caring. Additionally, five skills and eighteen behaviors should be nurtured in leaders based on several workshops with Saudi healthcare experts after the revision of organizational psychologists. As a result, every program that was developed has been built on this model.
Workforce Planning Theme	Importance of planning strategies sub-theme	Healthcare workforce planning aims to provide the ideal workforce to ensure optimum patient care in terms of quality, timing, place, skills, and even cost. With increasing future healthcare demands and the adoption of health transformation strategies, the importance of proper workforce planning increases despite its complexity.
	Examples of adopted strategies sub-theme	At present, there is coordination between the Vision Realization Office, Ministry of Health, health loading company, and Public Health Accreditation Board, and the SCFHS has been established to co-develop a program or plan strategy for the Health Transformation Vision 2030. Moreover, there are comprehensive plans to create governance for undergraduate education as an alignment between the Ministry of Education and Ministry of Health to enhance education and achieve the needs of the healthcare sector.
	Areas of planning that require exploration sub-theme	An emphasis on exploring and delineating the description of each healthcare worker must be given to ensure optimal distribution of efforts and skills. Decreasing fragmentation between healthcare sectors and working on getting real-time data are other areas requiring exploration.
	The golden rule for workforce planning sub-theme	One of the main rules mentioned was the effective utilization of real-time data in planning. Defining the terminologies and the scope of work is also important.
	Priorities that determine the needs to meet the vision for the 2030 sub-theme	Priorities include appropriate workforce distribution, decreasing the percentage of ex-patriates in certain specialties, and closing the gap between Saudization and expatriates. Attention must be given to the status and the existence of any variability in comparison to benchmarks.
	The need for a change management strategy sub-theme	Change management according to John Kotter has emotional and situational perspectives and occurs in eight steps: developing urgency, building a guiding team, creating a vision, communicating for buy-in, enabling action, creating short-term wins, do not let up, and making it stick
	Workforce upskilling sub-theme	Ideally, healthcare must be a learning healthcare system where skills are gained with constant engagement in one’s duties. The new skills should be included in the curricula of medical colleges and incorporated into continuous professional development (CPD).
	Attractive health ecosystem to the workforce sub-theme	Appropriate financial and non-financial incentives can greatly impact the health eco-system. Incentives include respect, an appreciative environment, courage for advocacy, and understanding fatigue. Coordination and use of automated services between providers make services faster and easier. Clarifying the duties and responsibilities of healthcare workers in addition to proper payments, work environment protection, and proper training and education are also essential for a better healthcare ecosystem. The foreign workforce must also be taken into consideration by providing them with suita-ble clinical units and housing conditions. Currently, there exists a program that deals with stress, burnout, and academic difficulties, called Daem.
Healthcare Quality Theme	Ideal quality of healthcare sub-theme	One of the most prevailing pride flags in Saudi Arabia is the healthcare industry, and the vision and mission of the SCFHS are for the idealization of the quality of healthcare.
	The current state of satisfaction of healthcare quality sub-theme	The quality of healthcare in Saudi Arabia is generally decent. However, the current satisfaction and attitudes are complex. Indeed, satisfaction is a continuous process and can never reach climax since it correlates with precious human life.
	Engaging the workforce with the enhancement of quality to meet the vision for the 2030 sub-theme	The workforce is already engaged to improve quality in the form of reflective practice.
	The importance of technology sub-theme	Technology is an integral part of the transformation. Additionally, it helps in the predictability of the workforce and market when AI is used correctly. However, technology can be a double-edged sword: when used correctly, it can enhance the workforce, yet it could remove the human element, thus taking away empathy.

## Data Availability

The data collected for this research are available in the Research and Development Department of the Saudi Commission for the Health Specialties.
